# Variation among human, veterinary and environmental *Mycobacterium chelonae-abscessus *complex isolates observed using core genome phylogenomic analysis, targeted gene comparison, and anti-microbial susceptibility patterns

**DOI:** 10.1371/journal.pone.0214274

**Published:** 2019-03-25

**Authors:** Susan B. Fogelson, Alvin C. Camus, W. Walter Lorenz, Ravikiran Vasireddy, Sruthi Vasireddy, Terry Smith, Barbara A. Brown-Elliott, Richard J. Wallace, Nabeeh A. Hasan, Udo Reischl, Susan Sanchez

**Affiliations:** 1 University of Georgia, College of Veterinary Medicine, Department of Pathology, Athens, GA, United States of America; 2 University of Georgia, Institute of Bioinformatics, Athens, GA, United States of America; 3 University of Texas Health Science Center at Tyler, Mycobacteria/Nocardia Research Laboratory, Department of Microbiology, Tyler, TX, United States of America; 4 Center for Genes, Environment and Health, National Jewish Health, Denver, CO, United States of America; 5 Institute of Clinical Microbiology and Hygiene, University Hospital of Regensburg, Regensburg, Germany; 6 University of Georgia, College of Veterinary Medicine, Department of Infectious Diseases, Athens, GA, United States of America; National Institute of Infectious Diseases, JAPAN

## Abstract

*Mycobacterium chelonae* is a member of the *Mycobacterium chelonae-abscessus* complex and a cause of opportunistic disease in fish, reptiles, birds, and mammals including humans. Isolates in the complex are often difficult to identify and have differing antimicrobial susceptibilities. Thirty-one previously identified rapidly-growing, non-tuberculous *Mycobacterium* sp. isolates cultured from biofilms, fish, reptiles, mammals, including humans, and three ATCC reference strains were evaluated with nine *M*. *chelonae-abscessus* complex whole genome sequences from GenBank by phylogenomic analysis, targeted gene comparisons, and *in-vitro* antimicrobial susceptibility patterns to assess strain variation among isolates from different sources. Results revealed minimal genetic variation among the *M*. *chelonae* strains. However, the core genomic alignment and SNP pattern of the complete 16S rRNA sequence clearly separated the turtle type strain ATCC 35752^T^ from the clinical isolates and human reference strain “*M*. *chelonae* chemovar *niacinogenes*” ATCC 19237, providing evidence of two distinct subspecies. Concatenation of the partial *rpoB* (752 bp) and complete *hsp65* (1,626 bp) sequence produced the same species/subspecies delineations as the core phylogeny. Partial *rpoB* and *hsp65* sequences identified all the clinical isolates to the appropriate species level when respective cut-offs of 98% and 98.4% identity to the *M*. *chelonae* type strain ATCC 35752^T^ were employed. The human strain, ATCC19237, was the most representative strain for the evaluated human, veterinary, and environmental strains. Additionally, two isolates were identified as *Mycobacterium saopaulense*, its first identification in a non-fish or non-human host.

## Introduction

*Mycobacterium chelonae* is a nontuberculous mycobacteria (NTM) within the *Mycobacterium chelonae-abscessus* complex, which also includes the closely related *Mycobacterium abscessus* subspecies *abscessus*, *Mycobacterium immunogenum*, *Mycobacterium salmoniphilum*, *Mycobacterium franklinii*, and *Mycobacterium saopaulense* [1–4]. Individual members cause disease in fish, reptiles, birds, and mammals, including humans [5–7]. Due to their phenotypic, biochemical, and genetic similarity, species identification can be problematic.

*M*. *chelonae-abscessus* complex members have been identified in municipal water supplies, soil, and biofilms, and cases of mycobacteriosis have been linked to environmental sources [8–10]. Zoonotic disease is also a significant concern [11, 12]. Although considered an opportunistic pathogen, *M*. *chelonae*, is being increasingly reported in both healthy and immune deficient human patients [13, 14]. *M*. *chelonae* is similarly concerning to the veterinary community, especially in aquatic species such as fish. Susceptibility varies among families of fish, but a link has also been made between disease and immune system compromise [15–17]. Highly dependent on correct identification, treatment regimens for *M*. *chelonae* infections exist for human patients, while effective treatments for fish are largely non-existent.

Accurate identification of *M*. *chelonae* poses a challenge to human and veterinary diagnostic laboratories. Reliability has improved as identification methods have evolved from biochemical testing to molecular typing, restriction fragment length polymorphism analysis of *hsp65* (*hsp65* PRA), DNA strip assays, and matrix-assisted ionization time of flight mass spectrometry (MALDI-TOF) [18, 19]. However, ambiguity remains due to deficiencies in public databases, inconsistencies in restriction patterns for *hsp65* PRA gel electrophoresis versus *in-silico* analysis, and a lack of consensus among laboratories regarding percent identity breakpoints used to differentiate closely related species [20].

In recent years, decreasing costs and increasing availability of molecular tools has enabled labs to investigate *M*. *chelonae-abscessus* complex isolates by whole genome sequencing (WGS) and target the most reliable genes for identification purposes [3, 10, 19]. While 16S rRNA gene sequencing is useful for identifying NTM isolates [21], partial 16S rRNA sequencing fails to separate *M*. *chelonae* and *M*. *abscessus* subsp. *abscessus* [22–24]. Other genes purported to differentiate closely related bacterial species include regions 3 and 5 of the β-subunit of the RNA polymerase gene (*rpoB)*, the Telenti sequence of the 65 kDa heat shock protein gene (*hsp65*), DNA gyrase subunits A (*gyr A)* and B (*gyr B*), translation elongation factor *Tu* (EF-*Tu*), manganese dependent superoxide dismutase (*Mn*-*SodA*), *Escherichia coli* secretion gene (*SecA*), and the 16S-23S internal transcribed spacer region (ITS) [24–27]. However, the diagnostic utility of many of these genes has not been evaluated for the *M*. *chelonae-abscessus* complex. At present, diagnostic laboratories employ a combination of gene targets to identify closely related species. The Nocardia/Mycobacteria Research Laboratory (Tyler, TX) uses targeted sequencing of *erm(41)* and *rpoB*, but uncertainty remains for *M*. *chelonae* isolates, as breakpoints for *rpoB* have not been established [28]. Many laboratories simply identify isolates to the *M*. *chelonae-abscessus* complex level [29]. This poses a risk to patients, as antibiotic susceptibilities vary among members of the complex [28, 30, 31].

Reports describe *M*. *chelonae* infections in individual hosts and epizootics within the same species [32–34]. Yet, little is known regarding strain variability among different animal species and the environment. In this study, a One Health approach investigating the genetic variation among 31 rapidly-growing *Mycobacterium* sp. isolates from biofilms, humans, diseased animals, and three ATCC reference strains were compared following WGS and core genome extraction. Isolates were evaluated by core phylogenomic analysis, targeted gene sequence phylogenetic analysis, *hsp65* PRA, in-silico dDNA-DNA hybridization, and antimicrobial minimum inhibitory concentration (MIC) determination. Results provide insight into strain variation between sources and the basis for a standard method for *M*. *chelonae* identification.

## Materials and methods

### Sample preparation

The analysis included 31 isolates previously identified as *M*. *chelonae* or *Mycobacterium* sp. from biofilms, fish, reptiles, and mammals, including humans, from the United States and Puerto Rico supplied by the Athens Veterinary Diagnostic Laboratory and the Mycobacteria/Nocardia Research Laboratory (MNRL), as well as three American Type Culture Collection (ATCC) reference strains (Table 1). Genomic DNA was extracted from Middlebrook 7H11 grown cultures using the UltraClean Microbial DNA Isolation Kit (Mo Bio Laboratories, Inc, Carlsbad, CA) following the manufacturer’s protocol. Approximately 15–28 ng/μL of DNA was submitted from each isolate to the Georgia Genomics Facility (The University of Georgia, Athens, GA) for DNA library preparation using Illumina TruSeq adaptors. Paired end (PE) 300-base reads were generated on an Illumina MiSeq PE300 sequencer (Illumina Inc., San Diego, CA).

**Table 1:** Mycobacterium chelonae-abscessus sequenced isolates.

Isolate	Host species	Tissue origin	Geographic location	Original identification method	Original identification	WGS identification
ATCC 19977^T^	*Homo sapiens*	soft tissue (knee)	Missouri	phenotyping/hybridization	*M*. *abscessus*	*M*. *abscessus*
ATCC 35752^T^	*Chelona corticata*	lung	Germany	phenotyping	*M*. *chelonae*	*M*. *chelonae*
ATCC 19237	*Homo sapiens*	gastric lavage	Germany	phenotyping/hybridization	*M*. *chelonae*	*M*. *chelonae*
seakrait	*Laticauda columbrina*	NA	Texas	phenotyping/*hsp65*	*M*. *chelonae*	*M*. *abscessus*
cichlid	Freshwater Cichlidae	spleen	Georgia	16S rRNA	*M*. *chelonae*	*M*. *chelonae*
pipefish	*Syngnathoides biaculeatus*	ovary	South Carolina	*hsp65* PRA	*Mycobacterium* sp.	*New species*
seahorse 1	*Hippocampus erectus*	tail	Georgia	*hsp65* PRA	*Mycobacterium* sp.	*New species*
seahorse 2	*Hippocampus erectus*	skeletal muscle	Georgia	*hsp65* PRA	*M*. *chelonae*	*M*. *chelonae*
seahorse 3	*Hippocampus whitei*	tail	Georgia	*hsp65* PRA	*M*. *chelonae*	*M*. *chelonae*
seahorse 4	*Hippocampus erectus*	ovary	Georgia	*hsp65* PRA	*M*. *chelonae*	*M*. *chelonae*
seahorse 5	*Hippocampus reidi*	ovary	Georgia	*hsp65* PRA	*Mycobacrterium* sp.	*M*. *chelonae*
seadragon 1	*Phyllopteryx taeniolatus*	soft tissue	Georgia	16S rRNA	*M*. *chelonae*	*M*. *chelonae*
seadragon 2	*Phycodurus eques*	liver/mesentery	Georgia	16S rRNA	*M*. *chelonae*	*M*. *chelonae*
trumpetfish	*Aulostomus maculatus*	soft tissue	South Carolina	phenotyping/*hsp65* PRA	*M*. *chelonae*	*M*. *chelonae*
turtle	*Platystemon megacephalum*	NA	Maryland	phenotyping/*hsp65* PRA	*M*. *chelonae*	*M*. *saopaulense*
python	*Morelia boeleni*	NA	Ohio	phenotyping/*hsp65* PRA	*M*. *chelonae*	*M*. *chelonae*
biofilm 1	Biofilm	aquarium system	Georgia	*hsp65* PRA	*M*. *chelonae*	*M*. *chelonae*
biofilm 2	Biofilm	aquarium system	Georgia	*hsp65* PRA	*M*. *chelonae*	*M*. *chelonae*
biofilm 3	Biofilm	aquarium system	Georgia	*hsp65* PRA	*M*. *chelonae*	*M*. *chelonae*
cow	*Bos taurus*	NA	Puerto Rico	phenotyping/*hsp65* PRA	*M*. *chelonae*	*M*. *saopaulense*
H7	*Homo sapiens*	sputum	Texas	*hsp65* PRA	*M*. *chelonae*	*M*. *chelonae*
H8	*Homo sapiens*	soft tissue (nasal)	North Carolina	*hsp65* PRA	*M*. *chelonae*	*M*. *chelonae*
H9	*Homo sapiens*	soft tissue (calf)	Massachusetts	*hsp65* PRA	*M*. *franklinii*	*M*. *franklinii*
H10	*Homo sapiens*	soft tissue (foot)	Minnesota	*hsp65* PRA	*M*. *chelonae*	*M*. *chelonae*
H11	*Homo sapiens*	sputum	Texas	*rpo*B	*M*. *chelonae*	*M*. *chelonae*
H12	*Homo sapiens*	soft tissue (axilla)	Kansas	*rpo*B	*M*. *chelonae*	*M*. *chelonae*
H13	*Homo sapiens*	eye	Massachusetts	*rpo*B	*M*. *chelonae*	*M*. *chelonae*
H14	*Homo sapiens*	synovial fluid (knee)	North Carolina	*rpo*B	*M*. *chelonae*	*M*. *chelonae*
H15	*Homo sapiens*	soft issue (finger)	North Carolina	*rpo*B	*M*. *chelonae*	*M*. *chelonae*
H16	*Homo sapiens*	sputum	California	*rpo*B	*M*. *chelonae*	*M*. *chelonae*
H17	*Homo sapiens*	soft tissue (leg)	California	*hsp65* PRA	*M*. *chelonae*	*M*. *chelonae*
H18	*Homo sapiens*	soft tissue (skin)	Massachusetts	*hsp65* PRA	*M*. *chelonae*	*M*. *chelonae*
H19	*Homo sapiens*	soft tissue (leg)	Ohio	*hsp65* PRA	*M*. *chelonae*	*M*. *chelonae*
H20	*Homo sapiens*	NA	Maryland	*hsp65* PRA	*M*. *chelonae*	*M*. *chelonae*

NA, Not Available; *hsp65*PRA, *hsp65* PCR-restriction enzyme analysis; WGS, whole genome sequencing

### Sequence preparation and assembly

Sequence read quality was assessed using FastQC [35]. Raw reads were trimmed using Trimmomatic software [36] run with the following settings: ILLUMINACLIP:TruSeq3-PE.fa:2:30:10 LEADING:20 TRAILING:10 SLIDINGWINDOW:4:20 MINLEN:50. Draft level genomes were assembled from trimmed reads using SPAdes software (version 3.6.2) [37]. Assembly metrics were evaluated using the Quality Assessment Tool for Genome Assemblies (QUAST) [38]. Automated genome annotation was performed using the RAST (Rapid Annotations using Subsystems Technology) server [39].

### Core genome alignment and phylogenomic analysis

A pair-wise genome content distance matrix was produced for the WGS assemblies of the 31 samples, three reference strains, and nine sequences in GenBank: *M*. *chelonae* ATCC 35752^T^ (turtle), *M*. *abscessus* subspecies *abscessus* ATCC 19977^T^ (human), *M*. *abscessus* subsp. *massiliense* CCUG48898 (human), *M*. *abscessus* subsp. *bolletii* MC1518 (human), *M*. *chelonae 1518* (human), *M*. *franklinii* DSM 45524^T^ (human), *M*. *immunogenum CCUG 47286*^T^ (drinking water), *M*. *salmoniphilum* ATCC 13758^T^ (chinook salmon), *M*. *saopaulense* EPM 10906^T^ using Progressive Mauve aligner [40]. Extraction of a core genome containing genes present in all 43 whole genomes was performed and the genes were concatenated using a custom perl script. Two outliers were identified and removed to perform core sequence analysis of the remaining 41 genomes. Phylogenomic analysis of a 3,204,105 bp core sequence, composed of 3,141 annotated regions, was performed to assess phylogenomic position using RAxML, employing GTR Gamma rapid bootstrapping and search for best scoring Maximum Likelihood model with 1000 bootstrap replications [41].

### Sequence analyses and phylogenetic comparisons

All assembled and annotated genomes were imported into Geneious for in-silico targeted gene evaluation [42]. Keyword searches identified genes of interest whose DNA sequences were then extracted from the annotated genomes. For the partial *rpoB* (752 bp), partial *hsp65* (441 bp), and partial ITS (245–257 bp), published primers were utilized in-silico [9, 18, 24, 43]. A multisequence nucleotide alignment for 16S rRNA (1,526 bp), *rpoB* (752 bp), *hsp65* (1,626 bp), *hsp65* (441 bp), *gyrA* (2,118 bp), *gyrB* (1,935–2,013 bp), EF-*Tu* (1,259 bp), Mn *sodA* (624 bp), *rec*A (1,041 bp), ITS (245–257 bp), and *erm*(41) (673 bp) was performed and percent identity between sequences achieved using default settings in the MUSCLE program with a maximum of 10 iterations [44]. GenBank sequences for *M*. *abscessus* subsp. *abscessus* ATCC 19977^T^, *M*. *chelonae* ATCC 35752^T^, *M*. *abscessus* subsp *massiliense* CCUG 48898, *M*. *franklinii* DSM 45524 or D16R27, *M*. *saopaulense* EPM 10906, *M*. *salmoniphilum* ATCC 13758, and *M*. *immunogenum* CCUG 47286 were included for partial *rpoB*, partial *hsp65*, and ITS alignments when available.

The *rpoB*, *hsp65* (441 bp), and 16S rRNA (1,526 bp) loci were further evaluated by multisequence alignment with 22 *Mycobacterium* sp. clinical isolates from Nogueira et al. [19]. Furthermore, 170 human sequences contributed by the MNRL were included in evaluation of the sequences for potential sequevars by evaluation of single nucleotide polymorphisms (SNPs) in the 752 bp sequence. The *M*. *chelonae* ATCC 35752^T^ reference strain was designated as sequevar 1 and subsequent sequevars were identified by SNPs in relation to it. These sequences were then translated for evaluation of amino acid discrepancies at loci of nucleotide difference.

RAxML (version 7.2.8) was used to estimate phylogenies and produce phylogenetic comparison matrices [41]. Phylogenetic trees were obtained from DNA sequences by GTR Gamma rapid bootstrapping and search for best scoring Maximum Likelihood model with 1000 bootstrap replications. In addition, concatenated sequences, partial *hsp65* (441 bp) and *rpoB*, as well as the concatenated complete *hsp65* (1,626 bp) and *rpoB* (752 bp) were evaluated as described above and compared to the core genomic phylogeny for evaluation of potential for diagnostic use.

#### *Erm* (41)

All isolates were evaluated for presence of *erm*(41) by generating a custom BLAST database for each individual assembly followed by BLASTn using the 673 bp *erm*(41) GenBank *M*. *abscessus* subsp. *abscessus* ATCC 19977^T^ NC 010397 as a query sequence [45].

#### *hsp65* and PCR-restriction fragment length polymorphism analysis of *hsp65 (hsp65* PRA*)*

Extraction of the partial *hsp65* (441 bp) from the annotated genome assemblies was performed in-silico. Primers Tb11 and Tb12 [18] were used to identify and extract a 441 bp region of interest including flanking sequence. Primer sequences were included in the analysis as minor variation in primer binding areas of sequences did occur.

In-silico restriction length polymorphism analysis of the partial *hsp65* sequence was performed targeting restriction sites for enzymes *Bst*EII and *Hae*III. A virtual gel was used to evaluate fragments larger than 35 bp. Using an algorithm similar to Taylor et al. [46], additional reference *Mycobacterium* species (*M*. *abscessus* subsp. *bolletii* MC 1518, *M*. *abscessus* subsp. *massiliense* CCUG 48898, *M*. *franklinii* DSM 45524, *M*. *fortuitum* CT6, *M*. *immunogenum* CCUG 47286, *M*. *septicum* DSM 44393, *M*. *farcinogenes* DSM 43637, *M*. *salmoniphilum* ATCC 13758, and *M*. *saopaulense* EPM 10906*)* were selected for comparison to other closely related species. Fragments were also compared to sequences in the database contained by http://app.chuv.ch/prasite.

#### dDNA-DNA Hybridization

Whole genome assemblies of 31 samples, three reference strains, and seven GenBank sequences were submitted to the Genome to Genome distance calculator [47] using *M*. *chelonae* ATCC 35752^T^ and *M*. *chelonae* ATCC 19237 as reference isolates. Formula 2 (identities/HSP length) was used to calculate a digital DNA-DNA hybridization (dDDH) estimate using a GLM-based method.

#### Minimum inhibitory concentrations (MIC) and colony morphology

Antimicrobial susceptibility testing was performed for 30 isolates harvested from Middlebrook 7H11 plates using a Sensititre RAPMYCO panel (Thermofisher Thermo Scientific, Oakwood Village, OH), following Clinical and Laboratory Standards Institute recommendations [48]. Clarithromycin was evaluated on days 3 and 14 of incubation. Sensititre RAPMYCO uses a standard-ordered broth microdilution panel for susceptibility testing and previously established breakpoints for rapidly growing mycobacteria (RGM) [49, 50]. In addition, colony morphologies were recorded.

#### GenBank accessions

Accessions used: NC_010397 *M*. *abscessus* subsp. *abscesssus* ATCC 19977^T^, CP010946 *M*. *chelonae* ATCC 35752^T^, CP007220 *M*. *chelonae* CCUG 47445^T^, GCA_000523895.1 *M*. *chelonae* MC 1518, NZ_HG964481 *M*. *farcinogenes* DSM 43637, NZ_CP011269 *Mycobacterium fortuitum* CT6, GCA_002013895.1 *M*. *franklinii* CV002 DSM 45524^T^, AY550238 *M*. *fuerthensis* DSM 44567 (*hsp65* partial), NZ_CP011530 *M*. *immunogenum* CCUG 47286^T^, NZ_AP014547.1 *M*. *abscessus* subsp. *massiliense* CCUG 48898 ^T^, CP009613.1 *M*. *abscessus* subsp. *bolletii* MC1518, NZ_HG322951 *Mycobacterium septicum* DSM 44393, GCA_002086715.1 *M*. *saopaulense* EPM10906 ^T^, GCA_002013645.1 *M*. *salmoniphilum* ATCC 13758^T^. Sequences from Noguiera et al. [19]: (*hsp*65) KT779818, KT779821-KT779824, KT779826-KT779827, KT779844, (rpoB) KT779876, KT779879-KT779882, and KT 779884-KT 779885, KT 779887-KT779902, (16S rRNA) MAEQ00000000 *M*. *chelonae* 96–1705, MAER00000000 *M*. *chelonae* 96–1717, MAES00000000 *M*. *chelonae* 96–1720, MAET00000000 *M*. *chelonae* 96–1724, MAEU00000000 *M*. *chelonae* 96–1728, MAEV00000000 *M*. sp. D16R24, MAEP00000000 *M*. *franklinii* D16R27, MAEW00000000 *M*. sp. D16Q13, MAEX00000000 *M*. sp. D16Q14, MAEY00000000 M. sp. D16Q16, MAFS00000000 *M*. *franklinii* D16Q19, MAEZ00000000 *M*. sp. D16Q20, MAFA00000000 *M*. *chelonae* D16Q24, MAFB00000000 *M*. sp. D17A2, MAFC00000000 *M*. sp. D16R12, MAFD00000000 *M*. sp. D16R18, MAFE00000000 *M*. *salmoniphilum* D16Q15, MAFF00000000 *M*. *chelonae* D16R2, MAFG00000000 *M*. *chelonae* D16R3, MAFH00000000 *M*. *chelonae* D16R7, MAFI00000000 *M*. *chelonae* D16R9, MAFJ00000000 *M*. *chelonae* D16R10, MAFK00000000 *M*. *chelonae* D16R14, MAFL00000000 *M*. *chelonae* D16R19, MAFM00000000 *M*. *chelonae* D16R20, MAFN00000000 *M*. sp. 96–892. Thirty-four whole genome sequences from this study have been deposited in GenBank under Bioproject: PRJNA347845, Biosamples: SAMN05897971-SAMN05898003.

## Results

### Core genomic analysis

Phylogenetic comparison of isolates using core genes observed in all genomes separated and identified species within the *M*. *chelonae-abscessus* complex, as well as two outliers, seahorse1 and pipefish. The outliers were 99.4% identical to each other, but the closest reference strain, *M*. *chelonae* ATCC 35752^T^, shared only 75.1% identity. BLASTn searches of the NCBI database placed the two closest to NZ_CP011269.1 *Mycobacterium fortuitum* strain CT6 and CP009914.1 *Mycobacterium* sp. VKM Ac-1817D, with only 88% identity and were removed from further analysis. The core genomes of the remaining 41 strains produced a 3,204,105 bp in length sequence with 3,141 coding sequences (CDS). Of the CDS, 2,367 were confirmed by RAST as genes, 683 were hypothetical protein CDS, and the remaining 91 were probable CDS. Within the core CDS, 16S rRNA, *rpoB*, *hsp65* partial, *hsp65* whole, *gyrA*, EF-*Tu*, Mn-*sodA*, and *rec*A were present. The sequenced reference strain, *M*. *chelonae* ATCC 35752^T^, was100% identical to the GenBank strains *M*. *chelonae* ATCC 35752^T^. There was 100% identity between the reference strain *M*. *abscessus* subsp. *abscessus* ATCC 19977^T^, GenBank sequences *M*. *abscessus* subsp. *abscessus* ATCC 19977^T^, *M*. *abscessus* subsp. *bolletii* MC 1518, and *M*. *chelonae* 1518, demonstrating the presence of improper sequence designations in GenBank. Since the GenBank *M*. *chelonae* ATCC 35752^T^ and *M*. *abscessus* subsp. *abscessus* ATCC 19977^T^ downloaded sequences were identical to the sequenced isolates, hereafter, *M*. *chelonae* ATCC 35752^T^ and *M*. *abscessus* subsp. *abscessus* ATCC 19977^T^ will represent the sequenced and downloaded sequences for each strain.

**Fig 1:**
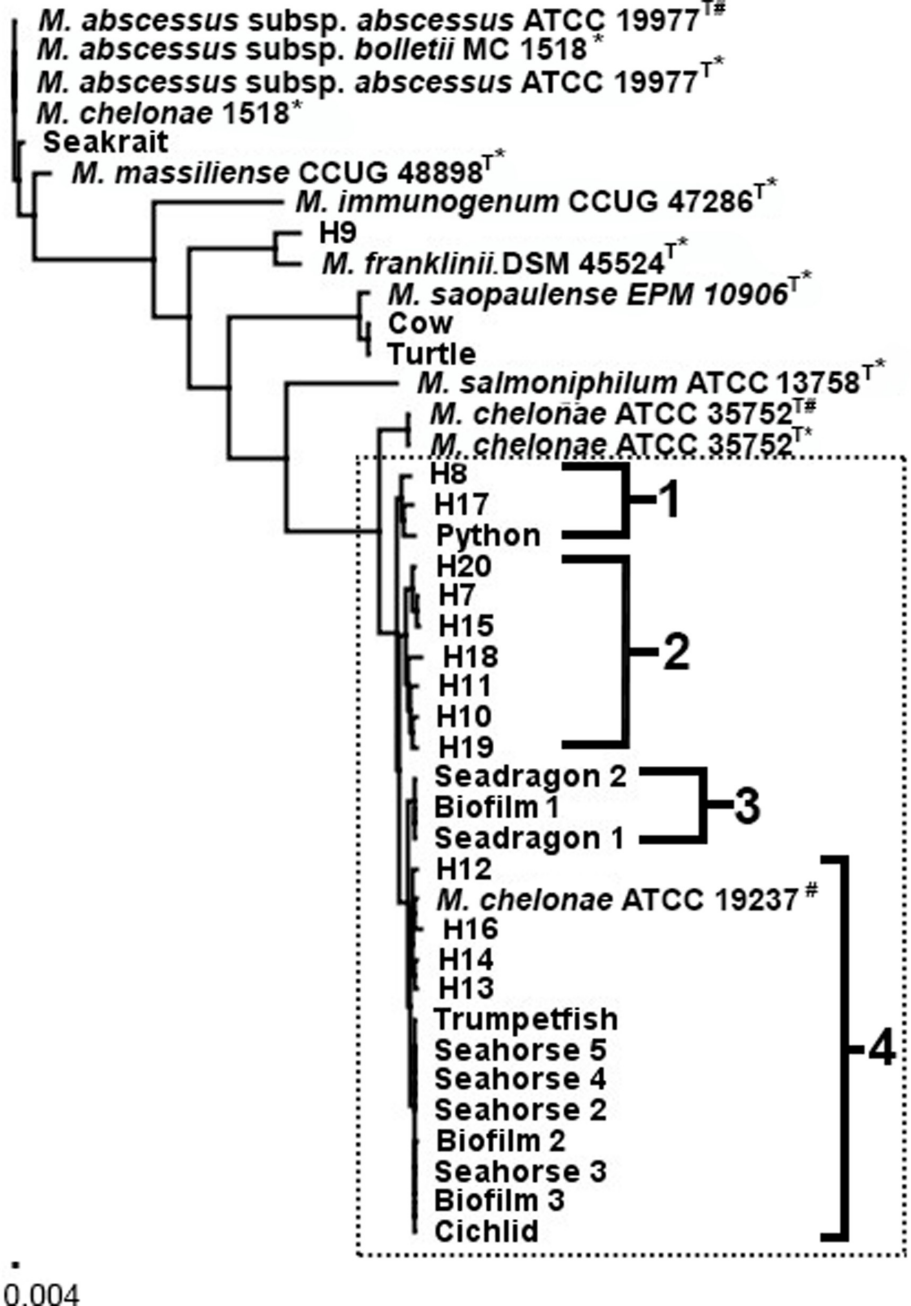
Phylogenomic comparison of *Mycobacterium chelonae-abscessus* isolates. Phylogenomic comparison of 32 *Mycobacterium* chelonae-abscessus. sequences relative to nine GenBank genome sequences using a core genome from all 41 sequences. Phylogeny was produced using the best scoring Maximum Likelihood model with 1000 bootstrap replications. Dotted box delineates *M*. *chelonae* clinical isolates clustered with “*M*. *chelonae* chemovar *niacinogenes*” ATCC 19237 and breakdown into 4 subclusters. Scale bar represents average number of nucleotide substitutions per site. 0.004 represents approximately 13,000 nucleotides that are not identical. ^T^ Denotes Type strain * Denotes sequence used from GenBank. ^#^ Denotes ATCC isolate sequenced in study.

Twenty-nine strains grouped closely with *M*. *chelonae* ATCC 35752^T^ using the core genomic comparison. However, four isolates were determined to be members of the *M*. *chelonae-abscessus* complex, but not *M*. *chelonae*. These isolates included seakrait, cow, turtle, and H9 (Fig 1).

Twenty-five of the 31 clinical isolates clustered with the sequenced *M*. *chelonae* ATCC 19237 with 98.4–99.6% identity (Fig 1). A mixture of human, fish, reptile, and biofilm isolates clustered in this large group, all with greater than 98.1% identity to each other. The current type strain *M*. *chelonae* ATCC 35752^T^ branched separately, with no greater than 96.5% identity to the 25 *M*. *chelonae* isolates. Minimal genetic variation was present within the isolates, although four distinct subclusters were present.

### Targeted gene analysis

Gene targets evaluated by multisequence alignment produced an identity matrix for comparison of sequences. Alignments of 16S rRNA, *gyrA*, *gyrB*, EF-*Tu*, *recA*, and Mn-*sodA* produced erroneous clustering or separation of the isolates and/or reference strains evidenced by inaccurate phylogenetic placement of the human isolates (EF-*Tu*, Mn-*sodA*, *gyrA*, *gyrB*) or lack of species separation (16S rRNA, *recA*) when compared to the core genomic results. Evaluation based on these alignments was not pursued further. However, the sequences for the clinical isolates and ATCC 19237 had at least three single nucleotide polymorphisms in the complete 16S rRNA sequence that distinctly separated them from the type strain ATCC 35752^T^ (S1 Fig and S1 Table). Furthermore, inclusion of 13 *M*. *chelonae* and 9 *M*. sp. isolates from Germany and Belgium revealed higher similarity to”*M*. *chelonae* chemovar *niacinogenes*” ATCC 19237 and *M*. *salmoniphilum* ATCC 13758^T^, respectively (S2 Fig).

#### ITS

A 257 bp ITS sequence was extracted for the *M*. *chelonae-abscessus* isolates. However, different ITS extraction product lengths were observed for isolate H9, *M salmoniphilum* ATCC 13758 (256 bp), *M immunogenum* CCUG 47286 (267 bp), and pipefish and seahorse1 (245 bp). Multi-sequence alignment of the clinical isolates and reference strains revealed adequate grouping into species-specific branches, but the high percent identity (99.1%) between H9 and the cow and turtle strains did not provide an accurate separation of the identities of the three isolates. For this study, isolates with greater than 98.8% (254/257bp) identity at the ITS locus to *M*. *chelonae* ATCC 35752^T^ were considered *M*. *chelonae* (S3 Fig).

#### hsp65

Targeted extraction of the 441bp partial *hsp65* gene sequence reproduced the main *M*. *chelonae* ATCC 35752^T^ clusters generated by core genome analysis (S4 Fig). Isolates with greater than 98.4% identity (434-441/441 bp) to *M*. *chelonae* ATCC 35752^T^ were considered *M*. *chelonae*. Although minimal sequence diversity is present at this locus (0–7 bp difference), two large sub-clusters, each containing strains 99.8–100% identical to each other are present. One sub-cluster contained exclusively human isolates (H7, H10, H11, H15, H18, H19, H20) and the other a mixture of *M*. *chelonae* ATCC 19237, human (H8, H12, H13, H14), fish (cichlid, trumpetfish, seadragon1, seadragon2, seahorse2, seahorse3, seahorse4, seahorse5), and biofilm (biofilm1, bioflm2, biofilm3) isolates. The partial *hsp65* sequence of human isolate H9 was 98.4% identical (434/441 bp) to *M*. *franklinii* DSM45524. The turtle and cow isolates also branched separately from the *M*. *chelonae* cluster and were 99.5% identical (439/441bp) to *M*. *saopaulense* EPM 10906. Inclusion of *M*. *chelonae* and *M*. sp. isolates from Nogueira et al. [19] showed a similar distribution where human *M*. *chelonae* isolates clustered together with 100% similarity to a mixture of environmental isolates, veterinary isolates, and “*M*. *chelonae* chemovar *niacinogenes*” ATCC 19237.

The complete 1,626 bp *hsp65* multisequence alignment was more discriminating than the partial sequence and produced some clusters mirroring the core genome phylogeny (S5 Fig). All isolates with greater than 95.3% identity (1,550/1,626 bp) to *M*. *chelonae* ATCC 35752^T^ at the complete *hsp65* were considered *M*. *chelonae*. As with the core genome and partial *hsp*65 phylogenies, the same group of human isolates branched together (H7, H10, H11, H15, H18, H19, H20) and shared 99.9–100% (1,625–1,626/1,626 bp) identity, but all *M*. *chelonae* isolates were greater than 99.1% identical to each other, showing minimal genetic variation in the group at this locus.

### rpoB

Phylogenetic analysis of *rpoB* (752 bp) produced similar phylogenetic positioning as the core genome (S6 Fig). Isolates with identities greater than 97.9% identity (736/752 bp) to *M*. *chelonae* ATCC 35752^T^ were considered as *M*. *chelonae*. The largest grouping consisted of multiple fish, biofilm, water, and human isolates, all of which had 99.9–100% identity to each other and contained ATCC 19237, but not ATCC 35752^T^.

One hundred and seventy *rpoB* sequences from the MNRL were evaluated with the 31 clinical isolates for SNPs, which ranged from zero in *M*. *chelonae* ATCC 35752^T^ up to 5 in some clinical isolates. Seventeen sequevars were recognized based on SNPs consistently identified at positions 24 (A-to-G), 36 (C-to-G), 90 (C-to-T), 99 (C-to-T), 100 (C-to-T), 102 (C-to-G), 123 (C-to-T), 126 (C-to-A), 204 (G-to-A), 237 (T-to-C), 363 (T-to-C), 384 (C-to-T), 385 (C-to-T), 430 (G-to-A), 444 (G-to-A), 480 (C-to-G), 559 (C-to-T), 654 (C-to-A), and 723 (G-to-T). However, sequence translations revealed only one amino acid change in a single human isolate from the sequence database, where a G-to-A substitution at codon 430 resulted in a glutamic acid substitution for lysine. Multisequence alignment of the additional *rpoB* sequences showed greater than 99.2% identity to *M*. *chelonae* ATCC 35752^T^.

### *hsp65* whole sequence and *rpoB*

Concatenation of partial *hsp65* (441 bp) and *rpoB* (752 bp) sequences produced a 1,193 bp sequence. The phylogenetic positioning of several isolates was not consistent with that of the core genome and no further analysis was performed. A concatenation of the complete *hsp65* (1,626 bp) and partial *rpoB* (752 bp) created a 2,378 bp sequence (S7 Fig). Clustering of clinical isolates was almost identical to the core genome phylogeny. However, unlike the core phylogeny, *M*. *chelonae* ATCC 35752^T^ branched at a different location. Isolates with greater than 96.1% (2,285/2,387 bp) identity to *M*. *chelonae* ATCC 35752^T^ were considered *M*. *chelonae*.

### 16S rRNA, *rpoB*, and partial *hsp65*

Concatenation of 16S rRNA (1,521–1,526 bp), *rpoB* (752 bp), and partial *hsp65* (441 bp) sequences from the present study and the Nogueira et al. [19] isolates revealed similar phylogenetic positioning to the core genome (S8 Fig). Human, veterinary, and environmental *M*. *chelonae* isolates grouped together with more than 97.2% similarity. However, *M*. *chelonae* ATCC 35752 and *M*. *chelonae* ATCC 19237 are 99.7% identical and grouped differently than the core phylogeny.

### *Erm* (41)

The erm (41) gene was only observed in GenBank reference strains *M*. *abscessus* subsp. *abscessus* ATCC 19977^T^, *M*. *chelonae* 1518, *M*. *abscessus* subsp. *bolletii* strain MC1518, and the seakrait isolate. All other clinical isolates and reference strains lacked this genetic sequence.

**Restriction fragment length polymorphism analysis (*hsp65* PRA)**. The partial 441 bp *hsp65* sequences were evaluated to produce two-step *Bst*EII and *Hae*III in-silico digestion reference patterns to compare the accuracy of identification in relation to the core genome phylogeny (S9 Fig) using fragments over 60 bp. In addition, fragments over 35 bp were also evaluated for pattern of fragmentation. *Bst*EII produced three groups, each with 2–4 fragments: 310/131 bp, 231/210 bp, and 231/116/84 bp. If these groupings are followed, *M*. *franklinii*, isolate H9 and *M*. *salmoniphilum* are considered within the grouping for *M*. *chelonae*. *Hae*III did not separate *M*. *salmoniphilum* from *M*. *chelonae* ATCC 35752^T^ unless fragments under 35 bp were considered. Additionally, human isolates H7, H10, H11, H15, H18, H19, and H20 were separated from other *M*. *chelonae* isolates. The patterns between these groups differ at 60 bp and under. The pattern for the *M*. *chelonae* 1518 GenBank sequence was the same as *M*. *abscessus* subsp. *abscessus* ATCC 19977^T^.

#### dDNA-DNA Hybridization

DNA-DNA relatedness for *M*. *chelonae-abscessus* members and clinical isolates were tested using *M*. *chelonae* ATCC 35752^T^ and “*M*. *chelonae* chemovar *niacinogenes”* ATCC 19237 as a reference (S2 Table). As expected, all *M*. *chelonae* isolates had a higher percent relatedness to *M*. *chelonae* ATCC 19237, ranging from 77.8% (CI 74.9–80.6%) to 95.7% (CI 94.2–96.8%), than to *M*. *chelonae* ATCC 35752^T^, which ranged from 63.3% (CI 60.4–66.1%) to 66.3% (CI 63.4–69.2%).

### MIC susceptibility and colony morphology

Twenty-seven non-genetically identical clinical isolates and three ATCC strains were evaluated using the Sensititre RAPMYCO panel (S3 Table). Subtle phenotypic differences in colony morphologies were observed when isolates were viewed simultaneously. The majority (22/30) were nonpigmented, smooth, glossy, and raised. The cow and turtle isolates produced similar colonies, but turned the 7H11 media brown after 7 days. The pipefish and seahorse1 outliers grew as nonpigmented, granular, glossy, raised, colonies, different from all others. Isolates H12, H13, H17, seahorse5 and python1 produced nonpigmented, rough, crusty, raised colonies.

MICs of the NTM isolates were classified as susceptible, intermediate, or resistant. A high degree of antimicrobial resistance was observed among all isolates, but the greatest resistance was found in the aquatic biofilm and fish isolates. However, 93% (28/30) were susceptible to the macrolide clarithromycin (S3 Table). Only *M*. *abscessus* subsp. *abscessus* ATCC 19977^T^ and isolate H10 were resistant to clarithromycin after 14 days. For the *M*. *chelonae* isolates, 70% (21/30 isolates) and 60% (18/30 isolates) were susceptible to the aminoglycosides tobramycin and amikacin, respectively. Only 50% of the *M*. *chelonae* isolates were susceptible to linezolid, the majority of which were of human origin (n = 9). Susceptibilities of *M*. *chelonae* were low for cefoxitin, trimethoprim/sulfamethoxide, imipenem, moxifloxicin, and ciprofloxacin at 3%, 10%, 3%, 13% and 20% (1/30, 3/30, 1/30, 4/30, 6/30), respectively. The human ATCC 19237 had a more resistant antimicrobial pattern than ATCC 35752^T^. The “*M*. *chelonae* chemovar *niacinogenes”* ATCC 19237 strain had a pattern more like the fish (cichlid, seahorse2, seahorse3, seahorse4, seahorse5, seadragon1), human (H10, H11, H12, H14, H17, H19, H20), and biofilm (biofilm1, biofilm2, biofilm3) isolates than ATCC 35752^T^.

## Discussion

Disease caused by members of the *M*. *chelonae-abscessus* complex in healthy and immunocompromised humans is increasing [14, 51–53]. *M*. *chelonae* infections are common in aquatic species and cause significant losses in certain groups of fish, particularly syngnathids (seahorses, seadragons and pipefish) [15, 54, 55]. Since *M*. *chelonae-abscessus* complex organisms are a human and veterinary health concern, characterization and appropriate identification methods are key to understanding the delicate balance of NTM interactions among humans, veterinary species, and the environment for disease control. Whole genome sequencing and core genome analysis was used to characterize NTM from fish, reptiles, mammals, and aquatic biofilms to investigate their genetic variation. High sequence homology was observed across *M*. *chelonae* isolates. Genetically similar strains infected a range of hosts and existed within environmental samples. A correlation between the environmental presence of *M*. *chelonae* and human disease has been established [56]. Similar strain characteristics and the low genetic variability of *M*. *chelonae* isolates from fish and biofilms suggests an environmental source of infection, a theory supported by a study of diseased pompano *Trachinotus carolinus* [12].

Certain human isolates tended to cluster using the different gene targeted sequencing methods, while others were more genetically similar to the aquatic animal or biofilm isolates. The consistent clustering of isolates H7, H10, H11, H15, H18-H20, suggests an epidemiologic link, although they share no known geographic or environmental associations. Human isolates H12, H13, H14, and H16 were genetically similar to fish and biofilm isolates, and to human “*M*. *chelonae* chemovar *niacnogenes”* ATCC 19237. It is reasonable to speculate that they may have originated from aquatic sources [57–59].

Core genomic comparison accurately identified closely related species in the *M*. *chelonae-abscessus* complex, as well as two divergent outliers (pipefish and seahorse1) cultured from syngnathid fish. Additional targeted gene sequencing, dDDH, and PRA analysis (S2 Table and S7 Fig) established the two outliers as a novel species, *Mycobacterium syngnathidarum* [60]. Core genome analysis of the remaining 41 whole mycobacterial genomes separated the human “*M*. *chelonae* chemovar *niacinogenes”* ATCC 19237 and turtle *M*. *chelonae* type strain ATCC 35752^T^ into subgroups. Clinical isolate sequences were more similar to ATCC 19237 (98.4–99.6%) than to ATCC 35752^T^ (96.5–96.6% identity). Adékambi et al. found similar results when comparing human clinical isolates with ATCC 19237 and ATCC 35752 ^T^ [61]. *M*. *chelonae* ATCC 35752^T^ also had a slightly different antimicrobial sensitivity profile than ATCC 19237 and the other *M*. *chelonae* isolates (S3 Table). Likewise, dDDH showed a difference in relatedness between the clinical isolates and *M*. *chelonae* ATCC 35752^T^. The genomic and antimicrobial data support recognition of two *M*. *chelonae* subspecies and indicate that use of *M*. *chelonae* ATCC 35752^T^ as a type strain may not be optimal for phylogenetic studies of *M*. *chelonae* isolates.

Core genome comparison revealed that earlier identification methods lacked fidelity for identification of *M*. *chelonae* isolates. Power of the core comparisons was high, because over half of the bacterial genome consisting of 4,898,027 bp and 4,489 CDS for *M*. *chelonae* ATCC 35752^T^ [62], was used for analysis. In the core alignment, 65.4% of the genome and 70% of the conserved coding regions were analyzed, including common housekeeping genes that are employed independently for species identification, such as EF-*Tu*, *SecA*, *gyrA*, Mn-*SodA*, 16S rRNA, *rpoB*, and *hsp65*. As a result, two human mycobacterial sequences in GenBank previously identified as *M*. *chelonae* 1518 and *M*. *abscessus* subsp. *abscessus* MC 1518 were found to be incorrect. The core alignment and presence of *erm* (41) delineate the sequences as *M*. *abscessus* subsp. *abscessus* ATCC 19977^T^. Isolates originally identified by *hsp65* or phenotyping as *M*. *chelonae* and *Mycobacterium* sp. (seahorse5, cow, turtle, and seakrait) were more precisely identified as *M*. *chelonae*, *M*. *saopaulense* and *M*. *abscessus* subsp. *abscessus*.

Similar to other published studies, WGS provided the greatest discrimination of *M*. *chelonae-abscessus* complex isolates, but is not yet practical in diagnostic settings where multilocus sequence analysis offers a practical alternative [10, 19, 63]. Comparison of commonly targeted genes to the core genome indicated that concatenated complete *hsp65* and partial *rpoB* sequences were diagnostically useful. Isolates with identities greater than 98.4% to turtle reference strain *M*. *chelonae* ATCC 35752^T^ were considered *M*. *chelonae*. While promising for species identification, there is no published data to support the proposed threshold and a larger sample size is needed to validate the method. Using the concatenated complete *hsp65* and partial *rpoB* sequences, the turtle type strain *M*. *chelonae* ATCC 35752^T^ and human reference strain *M*. *chelonae* ATCC 19237 both had greater than 99.1% identity to the main *M*. *chelonae* group of isolates, making differentiation between the potential subspecies difficult.

As previously reported, 16S rRNA analysis did not adequately differentiate species in the *M*. *chelonae-abscessus* complex [22] (S1 and S2 Figs and S1 Table). However, similar to that stated by Ballard et al. [64], SNPs patterns of the tested isolates designated *M*. *chelonae* were the same as ATCC 19237, not the turtle type strain ATCC 35752^T^ (three nucleotides different), further supporting the two as subspecies of *M*. *chelonae*. The genes *gyrA*, *gyrB*, EF-*Tu*, *RecA*, and Mn-*Sod* did not reliably identify species or produced inaccurate phylogenetic positioning, while the ITS, partial and complete *hsp65*, and *rpoB* loci were the most discriminating and identified isolates similarly to the core genomic analysis (S3, S4, S5 and S6 Figs). Partial *hsp65*, complete *hsp*65, and *rpoB* sequences identified the cow and turtle isolates as *M*. *saopaulense*, while *rpoB* and partial *hsp65* delineated H9 as *M*. *franklinii*. However, contradictory to the core genome analysis, *hsp65* (partial and complete), and the *rpoB* phylogenies, the ITS sequences of *M*. *salmoniphilum* ATCC 13758^T^ and H9 (*M*. *franklinii*) were 98.1% identical, which may not differentiate the species.

Regardless of phylogenetic differences produced by *hsp65* (partial and complete), partial *rpoB*, and the core genome, these methods can identify *M*. *chelonae* and closely related species when specified breakpoints are employed[19]. With other bacterial genera this is widely done for the16S rRNA locus where a 98.7% identity is applied as a cut-off level [65]. Breakpoints of 98.4% for partial *hsp65* (441 bp), 95.4% for complete *hsp65*, and 97.9% for *rpoB* or greater will identify *M*. *chelonae* when compared to the turtle type strain *M*. *chelonae* ATCC 35752^T^. Furthermore, inclusion of *M*. *chelonae* isolates from Germany and Belgium to the partial *hsp65* and *rpoB* analyses provides additional support for these breakpoints and the representative nature of ATCC 19237 to the current clinical isolates being evaluated worldwide, potentially making it a better candidate for comparison and identification purposes. Although a breakpoint was found for *hsp65*, additional partial and complete sequences are needed to confirm their validity.

Examination of a 170 sequence dataset provided by the Mycobacteria/Nocardia Research Laboratory confirmed the 97.9% *rpoB* breakpoint differentiates *M*. *chelonae* from other closely related species, but does not agree with Adékambi et al., which found intraspecies homology was 98.3–100% for the partial *rpoB* [24, 61, 66]. This discrepancy may be the result of comparison to *M*. *fortuitum* rather than *M*. *chelonae* strains in the earlier study. Further evaluation of SNPs from the *rpoB* sequences separated isolates into sequevars. Translation of the sequences confirmed that gene function was likely not affected, as amino acid sequences were unchanged in all but one sequence. Identifying *rpoB* sequevars may be useful for epidemiologic tracking of outbreaks, but no such connection could be made from the data set.

Replacement of PRA by targeted gene sequencing is supported by findings in this study. Comparisons of the partial *hsp65* PRA algorithm of Telenti et al. [18] and revised by Taylor et al. [46] and Chimera et al. [67] using in-silico digested fragments confirms the inability of PRA to differentiate species closely related to *M*. *chelonae*, likely a result of the greater discriminating power of “in-silico” analysis (1 bp) versus human interpretation of agarose gels (up to 10 bp). The fragments produced were 9–15 bp different than those derived using previously reported algorithms. For example, the PRA pattern for *M*. *chelonae* is 320/130 bp for *Bst*EII and 200/60/55 bp for *Hae*III, compared to the “in-silico” restriction pattern of 310/131 bp and 197/60/58/54 bp, respectively [46]. PRA analysis should not be used to identify mycobacteria in the *M*. *chelonae-abscessus* complex without revision of the algorithm to accommodate in-silico fragment sizes and fragments less than 60 bp in length, which were not assessed in the earlier studies that used traditional methods.

Susceptibility patterns, including significant antimicrobial resistance, have been reported for *Mycobacterium chelonae-abscessus* isolates and a multitude of acquired resistance mechanisms exist [31, 68–70]. One such example is the *MspA* gene, which, when expressed, has shown differential resistance of *M*. *chelonae* 9917 and *M*. *chelonae* ATCC 35752^T^ to rifampin (rifampicin), vancomycin, ciprofloxacin, clarithromycin, erythromycin, linezolid, and tetracycline. Investigation into specific resistance genes was not pursued for this study; however, the observed variable resistance to amikacin, ciprofloxacin, moxifloxacin, trimethoprim/sulfamethoxide, imipenem, cefoxitin, and linezolid among genetically similar isolates suggests differential expression of regulatory genes. The evaluated clinical isolates exhibited multidrug resistance, but biofilm isolates had the broadest resistance patterns [30, 49, 69]. Regardless of their origin, 96% of *M*. *chelonae* strains were susceptible to clarithromycin. Isolate H10 was resistant to clarithromycin and a gene mutation associated with resistance is suspected.

The *erm* (41) sequence in strains *M*. *abscessus* subsp. *abscessus* and *M*. *abscessus* subsp. *bolleti* MC 1518^T^, but not *M*. *chelonae*, can indicate inducible macrolide resistance [45, 71]. The presence of *erm* (41) in isolates originally identified as *M*. *chelonae* (*M*. *chelonae* 1518, *M*. *abscessus* subsp. *bolletii* MC1518, and seakrait), support their identification as *M*. *abscessus* subsp. *abscessus* by complete genome sequencing. Although *erm* (41) in a bacterial genome does not necessarily convey macrolide resistance, sensitivity to macrolides could serve as an aide in the identification of *M*. *chelonae-abscessus* complex species.

Colony morphology and phenotypic traits can aid conventional and molecular diagnostics [72, 73], but as demonstrated here, rarely provide sufficient evidence for definitive identification. Most isolates produced similar raised nonpigmented colonies that were smooth to dry and flaky, and virtually impossible to distinguish without side by side observation. Exceptions were the novel pipefish and seahorse1 isolates, which produced granular rough colonies, and *M*. *saopaulense*, which turned agar brown after several days of incubation [2]. This morphologic variance supported identification of the turtle and cow isolates as *M*. *saopaulense*, not *M*. *chelonae* as originally determined.

This whole genome evaluation of environmental, non-mammalian, and mammalian *M*. *chelonae-abscessus* isolates provides insight into the diversity of isolates within the complex and similarity of *M*. *chelonae* isolates. Identification of isolate similarity throughout different sources supports the necessity to understand the intricate relationship and interactions of the bacteria with humans, animals, and the environment. Especially because the high sequence homology among isolates from different geographic locations and host origin suggest an epidemiologic link. Core genome, dDDH, and 16S rRNA sequences indicate that *M*. *chelonae* is not a homogeneous species and that the current turtle type strain ATCC 35752 ^T^ and human ATCC 19237 represent two *M*. *chelonae* subspecies. Core genome comparison was the most discriminatory method for species identification, but concatenation of the complete *hsp65* and partial *rpoB* genes produced similar results and could be used for identification purposes.

## Supporting information

S1 Fig Phylogenetic comparison of *Mycobacterium chelonae-abscessus* isolates by 16S rRNA analysis. Phylogenetic comparison of *Mycobacterium chelonae-abscessus complex* isolates relative to eight GenBank sequences using the 16S rRNA 1,522 bp locus and two *M*. *syngnathidarum* outliers as an outgroup. Phylogeny was produced using the best scoring Maximum Likelihood model with 1000 bootstrap replications. Scale bar represents average number of nucleotide substitutions per site. 0.002 represents 2–3 nucleotides which are not identical.
T Denotes Type strain.
* Denotes sequence used from GenBank.
(TIF)

S2 Fig Phylogenetic comparison of *Mycobacterium chelonae-abscessus* isolates by 16S rRNA analysis. Phylogenetic comparison of *Mycobacterium chelonae-abscessus complex* isolates relative to eight GenBank sequences and sequences from Noguiera et al. (2007) using the 16S rRNA 1,522 bp locus and two *M*. *syngnathidarum* outliers as an outgroup. Phylogeny was produced using the best scoring Maximum Likelihood model with 1000 bootstrap replications. Scale bar represents average number of nucleotide substitutions per site. 0.002 represents 2–3 nucleotides which are not identical.
T Denotes Type strain.
* Denotes sequence used from GenBank.
(TIF)

S3 Fig Phylogenetic comparison of *Mycobacterium chelonae-abscessus* isolates by ITS analysis. Phylogenetic comparison of *Mycobacterium sp*. clinical isolates relative to eight reference sequences at the ITS locus using two *M*. *syngnathidarum* outliers as an outgroup. Phylogeny was produced using the best scoring Maximum Likelihood model with 1000 bootstrap replications. Dotted box delineates branch with M. chelonae isolates. Scale bar represents average number of nucleotide substitutions per site. 0.02 represents 0–1 nucleotides which are not identical.
T Denotes Type strain.
* Denotes sequence used from GenBank.
(TIF)

S4 Fig Phylogenetic comparison of *Mycobacterium chelonae-abscessus* isolates by partial *hsp65* analysis. Phylogenetic comparison of *Mycobacterium chelonae-abscessus* isolates including 22 *M*. sp. isolates from Belgium and Germany relative to eight GenBank sequences and two *M*. *syngnathidarum* outliers at the partial hsp65 441 bp locus. Phylogeny was produced using the best scoring Maximum Likelihood model with 1000 bootstrap replications. Scale bar represents average number of nucleotide substitutions per site. 0.02 represents 8–9 nucleotides which is not identical.
T Denotes Type strain.
* Denotes sequence used from GenBank.
(TIF)

S5 Fig Phylogenetic comparison of *Mycobacterium chelonae-abscessus* isolates by whole *hsp65* analysis. Phylogenetic comparison of *Mycobacterium chelonae*-abscessus isolates relative to eight GenBank sequences and two *M*. *syngnathidarum* outliers at the complete hsp65 1,626 bp locus. Phylogeny was produced using the best scoring Maximum Likelihood model with 1000 bootstrap replications. Dotted box delineates branch with M. chelonae and M. franklinii. Scale bar represents average number of nucleotide substitutions per site. 0.002 represents 3 nucleotides which are not identical.
T Denotes Type strain.
* Denotes sequence used from GenBank.
(TIF)

S6 Fig Phylogenetic comparison of *Mycobacterium chelonae-abscessus* isolates by partial *rpoB* analysis. Phylogenetic comparison of *Mycobacterium chelonae-abscessus* isolates relative to six reference strains and two *M*. *syngnathidarum* outliers at the partial rpoB 752 bp locus. Phylogeny was produced using the best scoring Maximum Likelihood model with 1000 bootstrap replications. Scale bar represents average number of nucleotide substitutions per site. 0.02 represents 15–17 nucleotides which are not identical.
^T^ Denotes Type strain.
* Denotes sequence used from GenBank.
(TIF)

S7 Fig Phylogenetic comparison of *Mycobacterium chelonae-abscessus* isolates by *rpoB* and *hsp65* analysis. Phylogenetic comparison of *Mycobacterium chelonae*-*abscessus* clinical isolates relative to seven reference sequences and two *M*. *syngnathidarum* outliers using the concatenated whole *hsp65* 1,626 bp and partial *rpoB* 752 bp sequences. Phylogeny was produced using the best scoring Maximum Likelihood model with 1000 bootstrap replications. Scale bar represents average number of nucleotide substitutions per site. 0.02 represents 34 nucleotides which are not identical.
T Denotes Type strain.
* Denotes sequence used from GenBank.
(TIF)

S8 Fig Phylogenetic comparison of *Mycobacterium chelonae-abscessus* isolates by 16S rRNA, *rpoB* and *hsp65* analysis. Phylogenetic comparison of *Mycobacterium chelonae*-*abscessus* clinical isolates from the USA, Belgium, and Germany relative to four GenBank sequences and two *M*. *syngnathidarum* outliers using the concatenated whole *hsp65* 1,626 bp, partial *rpoB* 752 bp, and partial *hsp65* 441 bp sequences. Phylogeny was produced using the best scoring Maximum Likelihood model with 1000 bootstrap replications. Scale bar represents average number of nucleotide substitutions per site. 0.02 represents 35 nucleotides which are not identical.
T Denotes Type strain.
* Denotes sequence used from GenBank.
(TIF)

S9 Fig Phylogenetic comparison of *Mycobacterium chelonae-abscessus* isolates by partial *hsp65* PRA analysis. Summary of in-silico PCR-restriction length polymorphism analysis results performed on the partial hsp65 (441 bp) fragment (hsp65 PRA). Results are arranged according to the Taylor et al. (63) algorithm with slight modification to account for fragment length created in-silico and inclusion of fragments 35 bp or greater.
T Denotes type strain.
(TIF)

S1 Table Nucleotide location substitution for whole 16S sequence of *M*. *chelonae* isolates. Delineation of sequevars identified within clinical isolates at 16S rRNA.
(XLSX)

S2 Table dDNA-DNA hybridization of M. *chelonae-abscessus* isolates and two *M*. *syngnathidarum* outliers. dDDH relatedness of clinical isolates compared to *M*. *chelonae* ATCC 35752^T^ and “*M*. *chelonae* chemovar *niacinogenes*” ATCC 19237. Confidence intervals are denoted within brackets.
(XLSX)

S3 Table Drug susceptibility data of Mycobacterium chelonae-abscessus clinical isolates reported as MICs.^a^ ^a^ Green shading represents susceptible (S); Green to yellow shading represents intermediate susceptible (I); Red to yellow shading represents intermediate resistant (I); Red shading represents resistant (R). Susceptibility patterns interpreted using CLSI recommendations. ^b^ NA, Not available. Isolates with missing Linezolid, Moxifloxacin, and Trimethoprim/Sulfamethoxazole values were evaluated for MIC prior to the use of these antibiotics.
^T^ Denotes type strain.
(XLSX)
