# Supplementary figures and images for: Variation among human, veterinary and environmental Mycobacterium chelonae-abscessus complex isolates observed using core genome phylogenomic analysis, targeted gene comparison, and anti-microbial susceptibility patterns
Source: PLoS One. 2019 Mar 25;14(3):e0214274. doi: 10.1371/journal.pone.0214274 (PMC6433289; doi:10.1371/journal.pone.0214274)

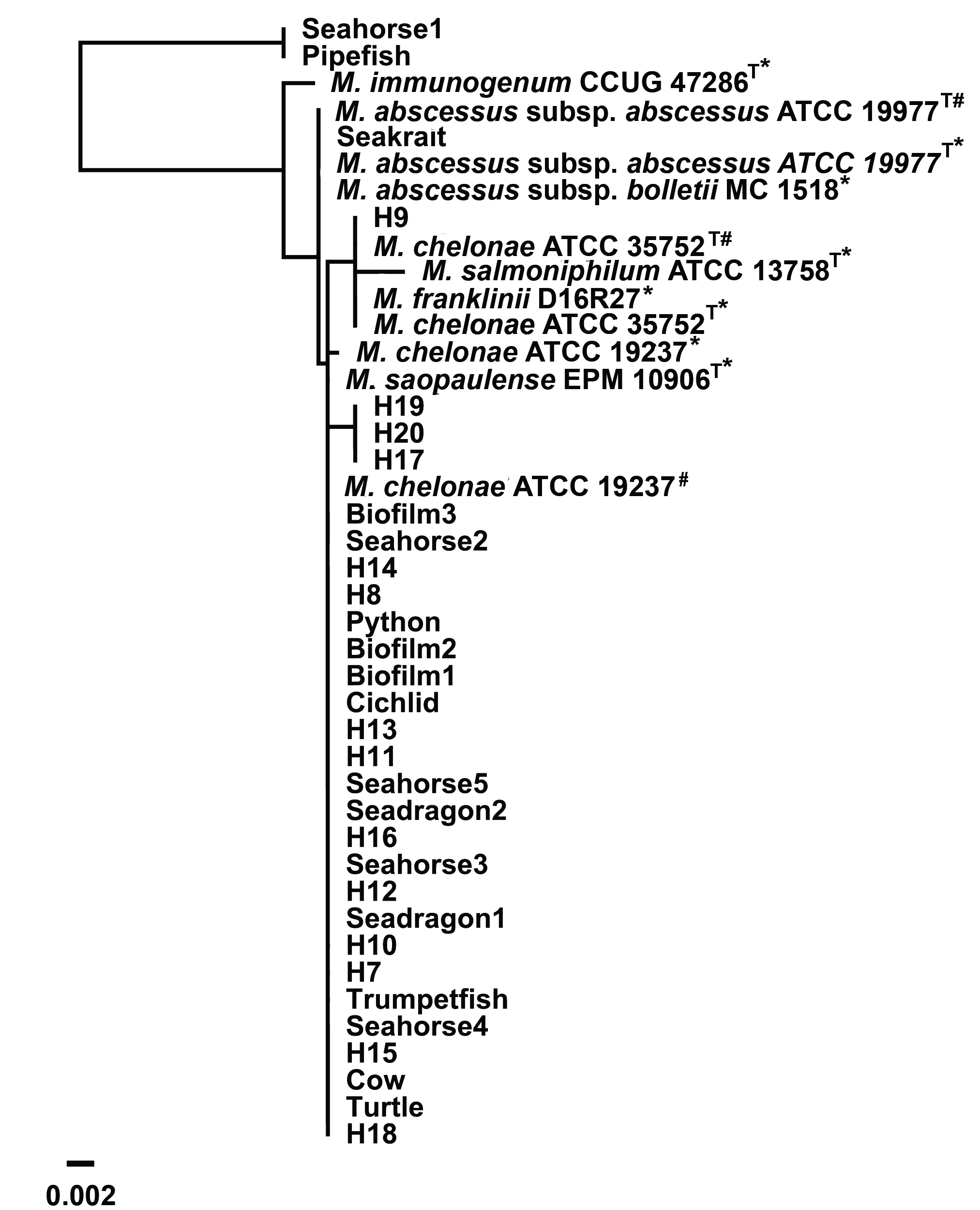

Supplement: S1 Fig — Phylogenetic comparison of Mycobacterium chelonae-abscessus complex isolates relative to eight GenBank sequences using the 16S rRNA 1,522 bp locus and two M. syngnathidarum outliers as an outgroup. Phylogeny was produced using the best scoring Maximum Likelihood model with 1000 bootstrap replications. Scale bar represents average number of nucleotide substitutions per site. 0.002 represents 2–3 nucleotides which are not identical. T Denotes Type strain. * Denotes sequence used from GenBank. (TIF) [file pone.0214274.s001.tif]

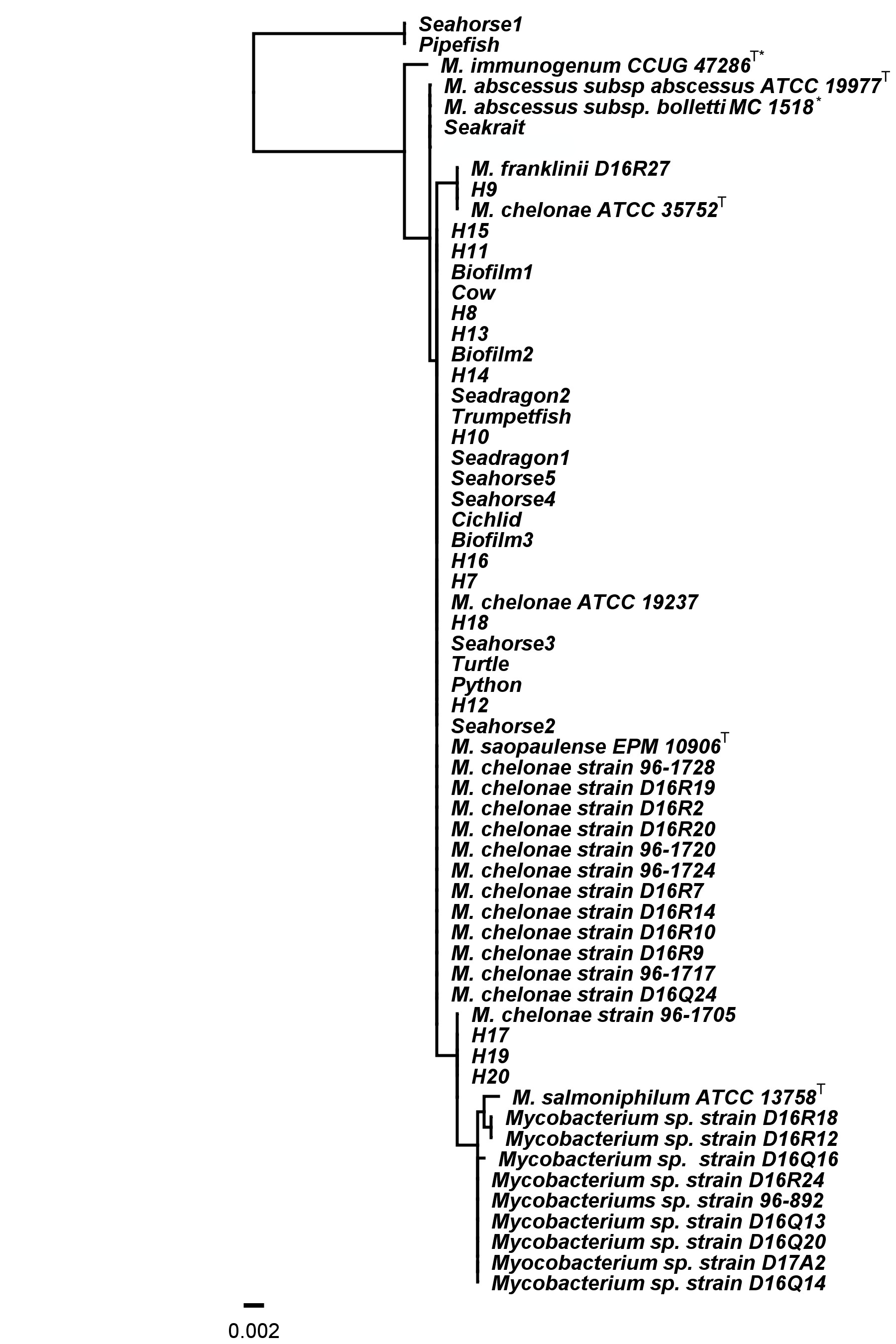

Supplement: S2 Fig — Phylogenetic comparison of Mycobacterium chelonae-abscessus complex isolates relative to eight GenBank sequences and sequences from Noguiera et al. (2007) using the 16S rRNA 1,522 bp locus and two M. syngnathidarum outliers as an outgroup. Phylogeny was produced using the best scoring Maximum Likelihood model with 1000 bootstrap replications. Scale bar represents average number of nucleotide substitutions per site. 0.002 represents 2–3 nucleotides which are not identical. T Denotes Type strain. * Denotes sequence used from GenBank. (TIF) [file pone.0214274.s002.tif]

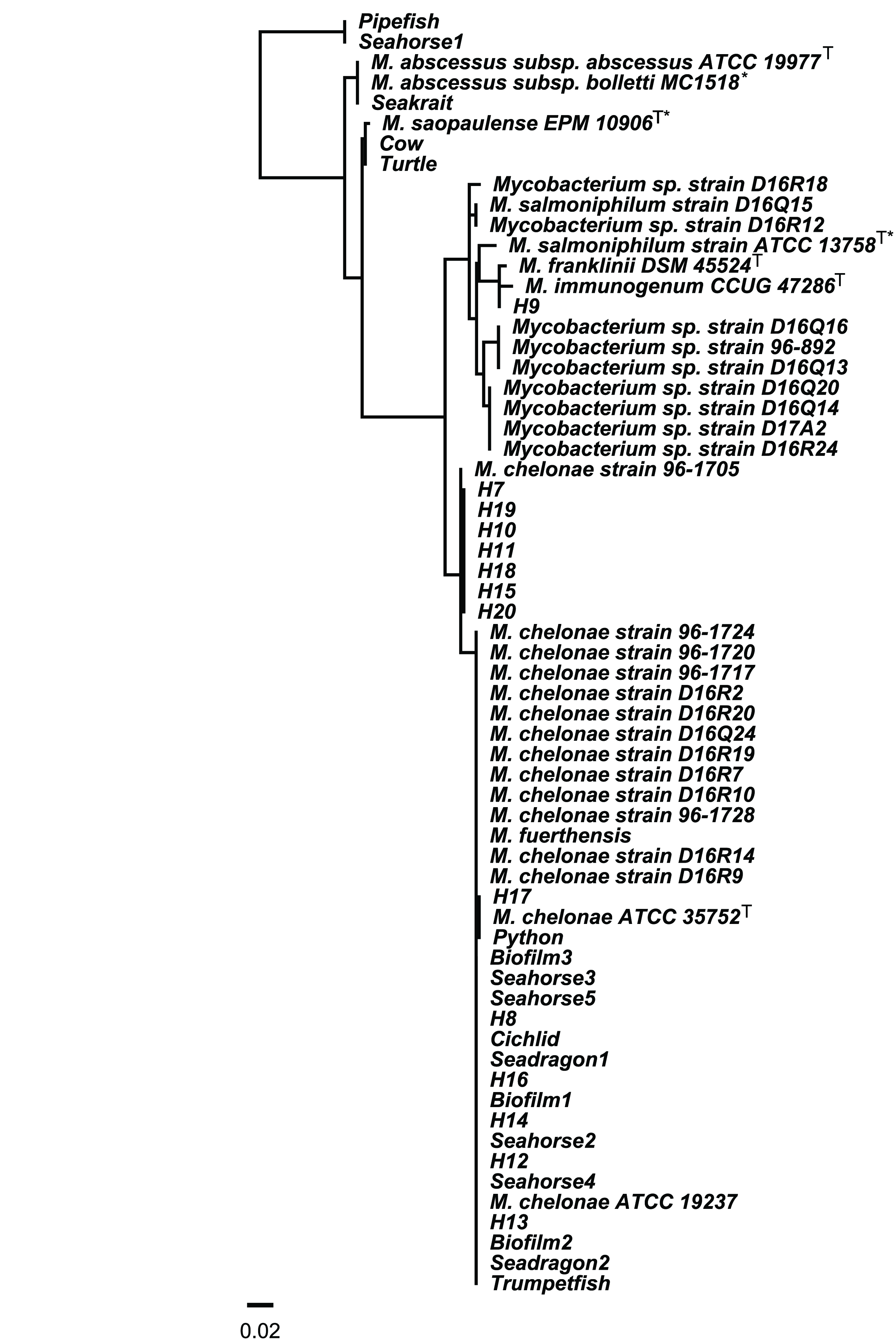

Supplement: S4 Fig — Phylogenetic comparison of Mycobacterium chelonae-abscessus isolates including 22 M. sp. isolates from Belgium and Germany relative to eight GenBank sequences and two M. syngnathidarum outliers at the partial hsp65 441 bp locus. Phylogeny was produced using the best scoring Maximum Likelihood model with 1000 bootstrap replications. Scale bar represents average number of nucleotide substitutions per site. 0.02 represents 8–9 nucleotides which is not identical. T Denotes Type strain. * Denotes sequence used from GenBank. (TIF) [file pone.0214274.s004.tif]

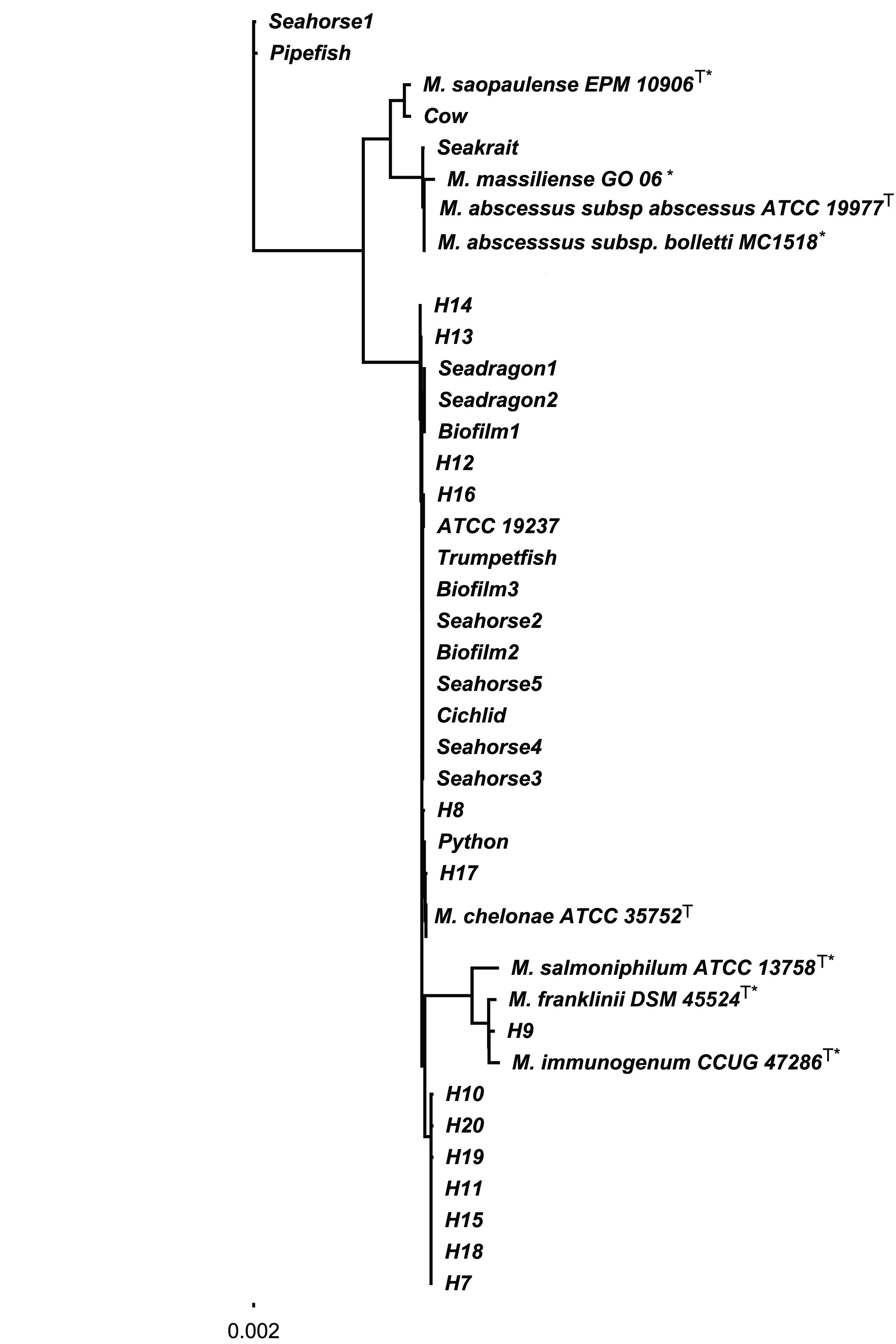

Supplement: S5 Fig — Phylogenetic comparison of Mycobacterium chelonae-abscessus isolates relative to eight GenBank sequences and two M. syngnathidarum outliers at the complete hsp65 1,626 bp locus. Phylogeny was produced using the best scoring Maximum Likelihood model with 1000 bootstrap replications. Dotted box delineates branch with M. chelonae and M. franklinii. Scale bar represents average number of nucleotide substitutions per site. 0.002 represents 3 nucleotides which are not identical. T Denotes Type strain. * Denotes sequence used from GenBank. (TIF) [file pone.0214274.s005.tif]

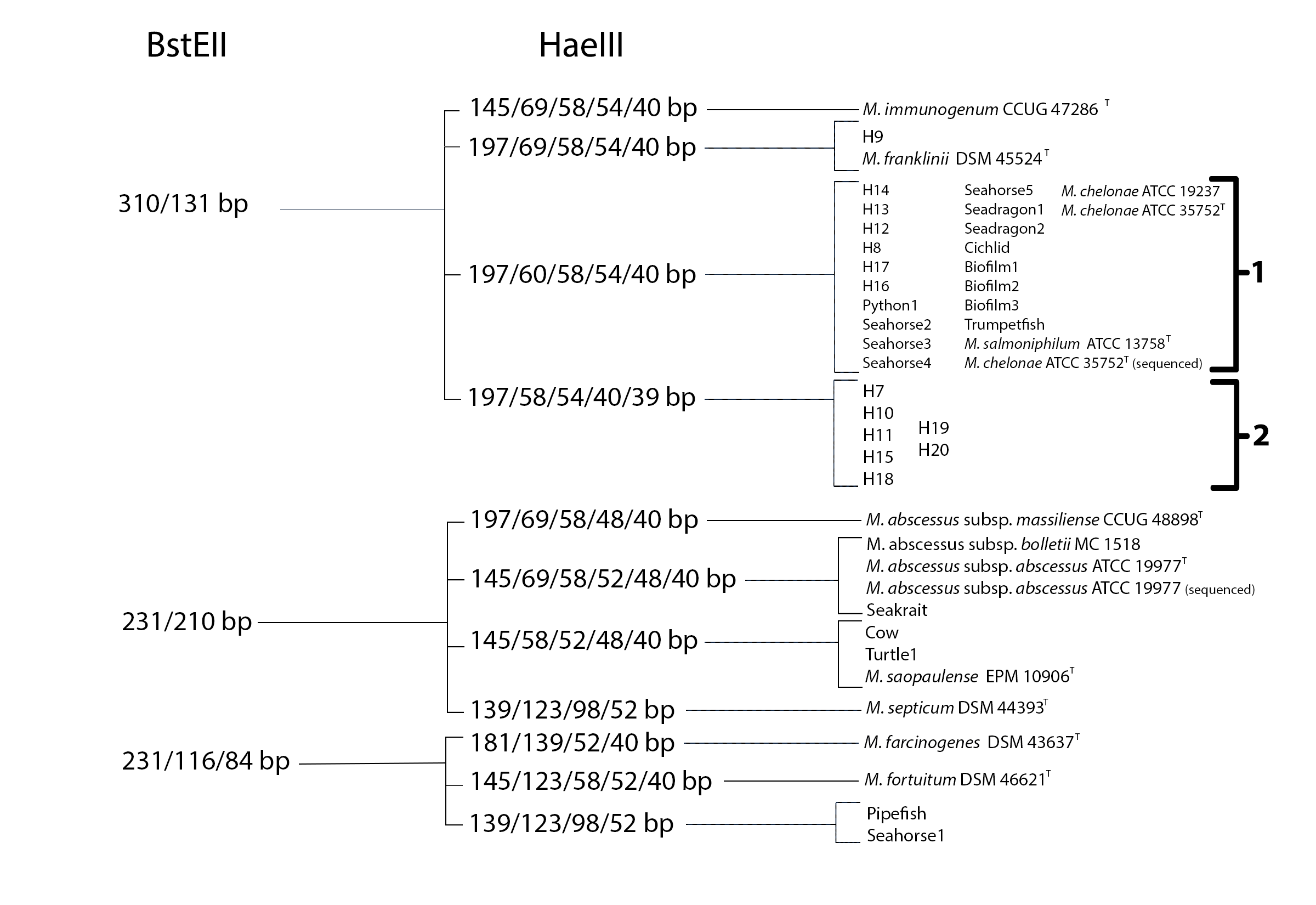

Supplement: S9 Fig — Summary of in-silico PCR-restriction length polymorphism analysis results performed on the partial hsp65 (441 bp) fragment (hsp65 PRA). Results are arranged according to the Taylor et al. (63) algorithm with slight modification to account for fragment length created in-silico and inclusion of fragments 35 bp or greater. T Denotes type strain. (TIF) [file pone.0214274.s009.tif]
